# Mortality due to COVID-19 in Spain and its association with environmental factors and determinants of health

**DOI:** 10.1186/s12302-022-00617-z

**Published:** 2022-04-26

**Authors:** Dante R. Culqui Lévano, Julio Díaz, Alejandro Blanco, José A. Lopez, Miguel A. Navas, Gerardo Sánchez-Martínez, M. Yolanda Luna, Beatriz Hervella, Fernando Belda, Cristina Linares

**Affiliations:** 1 Reference Unit On Climate Change, Health and Urban Environment National School of Health, Carlos III Health Institute, Monforte de Lemos 5, ZIP 28029 Madrid, Spain; 2 The UNEP DTU Partnership, Copenhagen, Denmark; 3 State Meteorological Agency (AEMET), Calle Rios Rosas, 44, Madrid, Spain

**Keywords:** COVID-19 mortality, Air pollution, Temperature, Humidity, Determinants of health

## Abstract

**Background:**

The objective of this study was to identify which air pollutants, atmospheric variables and health determinants could influence COVID-19 mortality in Spain. This study used information from 41 of the 52 provinces in Spain (from Feb. 1, to May 31, 2021). Generalized Linear Models (GLM) with Poisson link were carried out for the provinces, using the Rate of Mortality due to COVID-19 (**CM**) per 1,000,000 inhabitants as dependent variables, and average daily concentrations of PM_10_ and NO_2_ as independent variables. Meteorological variables included maximum daily temperature (*T*max) and average daily absolute humidity (HA). The GLM model controlled for trend, seasonalities and the autoregressive character of the series. Days with lags were established. The relative risk (RR) was calculated by increases of 10 g/m^3^ in PM_10_ and NO_2_ and by 1 ℃ in the case of *T*max and 1 g/m^3^ in the case of HA. Later, a linear regression was carried out that included the social determinants of health.

**Results:**

Statistically significant associations were found between PM_10_, NO_2_ and the **CM**. These associations had a positive value. In the case of temperature and humidity, the associations had a negative value. PM_10_ being the variable that showed greater association, with the **CM** followed of NO_2_ in the majority of provinces. Anyone of the health determinants considered, could explain the differential geographic behavior.

**Conclusions:**

The role of PM_10_ is worth highlighting, as the chemical air pollutant for which there was a greater number of provinces in which it was associated with **CM**. The role of the meteorological variables—temperature and HA—was much less compared to that of the air pollutants. None of the social determinants we proposed could explain the heterogeneous geographical distribution identified in this study.

**Supplementary Information:**

The online version contains supplementary material available at 10.1186/s12302-022-00617-z.

## Background

The corona virus pandemic (COVID-19) has become the principal public health issue of our time. According to daily mortality surveillance data [21], as of May 3, 2020, during the end of the first wave in Spain, there were an estimated 81,958 deaths due to COVID-19, and an excess mortality of 50 percent compared to the prior year. In early May of 2020, the World Health Organization (WHO) calculated an alarming rate of infection around the world, estimated at over 3.5 million infected patients [13]. As 26 of February 2022, a total of 10,977,524 confirmed cases of COVID-19 and 99,410 deaths have been reported in Spain [1].

Without a doubt, the COVID-19 pandemic poses questions for which there are still no answers, including the causes of disease, the factors that increase transmission, and the reasons behind its severity and mortality.

In general, the environment and air pollution in particular play an important role in the transmission, severity [39] and mortality [18] of some diseases, including COVID-19 [19, 38]. The adverse effects of environmental pollution on human health are widely recognized, and it has been shown that chronic exposure to air pollutants contributes to increases in hospitalizations and mortality, primarily related to cardiovascular system and respiratory problems, which can cause various diseases including cancer [32]. An analysis of The Lancet Commission on pollution and health suggested that air pollution is responsible for at least 16 percent of global deaths, and it is considered the primary cause of avoidable death in the world [35]. In general, poor air quality has also been linked to an increase in mortality due to Severe Acute Respiratory Syndrome (SARS) [14].

During the COVID-19 pandemic it has been observed that patients that become infected with SARS-CoV-2 often experience serious complications, including multi-organ failure, septic shock, pulmonary edema, severe pneumonia and respiratory stress, in many cases with fatal consequences [10]. Some authors consider that air pollution could contribute to the severity [7] and mortality [12] of COVID-19. However, the COVID-19 mortality rate (**CM**) during the first wave varied in different countries and depended, among other things, on the response capacity of countries [28]. It was also influenced by the characteristics of SARS-CoV-2, which has undergone mutations in different regions around the world [48]. This is a topic that has not been studied as extensively as has, for example, incidence and hospital admissions.

There is evidence that chronic exposure to air pollution increases respiratory and cardiovascular toxicity [24]. It has also been observed that particulate matter (PM) can act as a transporter for multiple infectious micro-organisms that have an impact on immunity and could increase susceptibility to different diseases [57]. Various research studies suggest that exposure to PM_2.5_ and PM_10_ increases the risk of COVID-19 infection [58], including a study in the United States (USA), which showed that long-term exposure to fine particulate matter (PM_2.5_) increases the risk of mortality due to COVID-19 [63]. Another study in Italy identified PM_10_ as the pollutant that presented the strongest correlation with the number of deaths due to COVID-19 [16], and a study in Spain also found evidence of an association between mortality due to COVID-19 and different air pollutants [38].

With respect to NO_2_, a study in the United Kingdom (UK) considered NO_2_ to be the primary contributor to increases in the number of deaths and the number of cases of COVID-19, above all during the early phases of the pandemic [58].

On the other hand, there are studies of SARS, carried out prior to the pandemic in Beijing, Hong Kong, Guangzhou and Taiwan, that suggest that the optimal environmental temperature for the survival of the virus is 16–28 °C. However, in the case of SARS-CoV-2, this evidence is not sufficient to identify the true role of temperature in the COVID-19 pandemic. Initially, the absence of a correlation between temperature and incidence of COVID-19 was attributed to methodological deficiencies and the fact that in many parts of the world the studies were carried out during the winter [42]. However, wider investigations in different climate zones and in different geographical areas around the world have not been able to clearly establish the role that temperature plays in COVID-19 incidence and mortality [6].

Relative humidity (indoor or outdoor) is considered an important factor in the severity of some respiratory diseases [65]. Some studies suggest that low air humidity may be an important risk factor for respiratory infections and could be responsible for an increase in rates of general mortality [5]. During the first wave of COVID-19, it was observed in Italy that a dry climate contributed to better transmission of SARS-CoV-2 [69], however, the evidence that relates COVID-19 mortality to humidity is scarce.

Many social and demographic determinants have been related to COVID-19 mortality. Poverty, economic circumstances and inequities in health services could contribute to increases in **CM** [45]. In the United States a relationship was found between access to health services [43] and **CM**, though race was not found to be associated with **CM** [3] Another study linked population density and lack of employment to high **CM** [53], and sex [46] and mobility were also included as important determinants of COVID-19 transmission [9], though there was no observed association with **CM**.

As mentioned in other studies, environmental factors act together [31] and therefore, they must be included together in analysis to determine the effect they have on COVID-19 mortality. Thus, the objective of this study was to identify which air pollutants, atmospheric variables and health determinants could influence COVID-19 mortality in Spain.

## Methods

An ecological, longitudinal retrospective time series study was carried out between February 1, and May 31, 2020 (first wave in Spain). The study included 41 of 52 Spanish provinces; not all provinces were studied due to difficulties in obtaining information for the desired study variables, as described in the inclusion criteria.

Inclusion criteria: the study included those provinces with available information on chemical air pollutants such as nitrogen dioxide (NO_2_), particulate matter (PM_10_) and meteorological variables such as average maximum temperature (*T*max) and average absolute humidity (HA). A maximum information loss of 10% was set for the days studied for all independent variables considered. In the event that there was more than 10% missing data for pollutants or meteorological variables in a province, that variable was not considered in the analysis for that province.

## Variables

### Independent variables

#### Air pollutants

Average daily concentrations of PM_10_ and NO_2_ in g/m^3^ were used, obtained from stations located in the different provinces analyzed [Source: Ministerio para la Transición Ecológica y el Reto Demográfico (MITECO)].

#### Meteorological variables

The daily values of maximum temperature (*T*max) were used as well as daily absolute humidity (HA) in g/m^3^, because they presented better behavior with the COVID-19 variables analyzed [38, 65] [Source: Agencia Estatal de Meteorología (AEMET)]. The meteorological variables were obtained from the representative observatory of each province according to AEMET. The values of air pollutants have been obtained as the average values of the set of stations located in each province.

HA was obtained based on average daily relative humidity (HR) and average daily temperature (*T*) [27]. Absolute humidity was calculated based on the Clausius–Clapeyron equation, as follows [30]:

$${\text{HA}} = \frac{{6.112 \times {\text{e}}^{[17.67 \times T/ + 243,5]} \times {\text{HR}} \times 2.1674}}{273,15 + T}.$$

### Health determinants

In order to explain the heterogeneity in the geographical distribution of the COVID-19 mortality results associated with air pollutants and meteorological variables, other health determinants were analyzed, grouped using the Lalonde–Laframboise epidemiological model [17]. This model classifies variables into groups related to human biology, lifestyles, environment and health services. Variables studied were:

- Lifestyles: considered through average mobility.
- Environment: included the physical, social and socioeconomic environment. The variables studied according to these characteristics are described here:
  - Physical environment: it includes the existence of an airport or not in each province; the annual average values of PM_2.5_, PM_10_ and NO_2_ in the period 2017–2019; the number of petrol cars per inhabitant; the number of diesel cars per inhabitant; the number of total cars excluding low and zero emission vehicles.
  - Social environment: including rurality and dwellings of less than 30 m^2^ (as a proxy for overcrowding).
  - Socio-economic environment: including income level, deprivation index and environmental expenditure.
- Health system responses:
  Among the variables corresponding to this group, the following were studied for each province: the number of consulting rooms; the number of health centres; the average number of ambulances per inhabitant; the average number of family doctors per inhabitant; the average number of nurses per inhabitant; the average number of doctors and nurses per inhabitant [11]; the number of beds per 1000 inhabitants; the number of ICU beds per 1000 inhabitants; the new contracts of health personnel (registered with the Social Security in the last year); the number of new contracts of health personnel (registered with the Social Security) during the last year. f.; the number of beds per 1000 inhabitants; the number of ICU beds per 1000 inhabitants and the new contracts of health personnel (registered with the Social Security) during the last year (for sources of information, see Additional file 1: Table S2).

### Dependent variables

The dependent variables were calculated based on the number of positive cases of COVID-19. Cases diagnosed as positive for COVID-19 were defined based on positive PCR test results in 99.74 percent of the data. The remaining cases were diagnosed based on presentation of symptoms compatible with the disease.

The rate of mortality due to COVID-19 (**CM**) per 1,000,000 inhabitants was used as the dependent variable, calculated in the following way:

Rate of mortality due to COVID-19 (**CM**) per 1,000,000 inhabitants: (number of COVID-19 positive deaths/population) × 1,000,000 inhabitants.

Information on deaths was provided by the National Epidemiology Center at the Carlos III Health Institute. Population data at the province level were provided by the National Institute of Statistics (INE). The dependent variables were calculated based on the number of positive cases of COVID-19.

#### Analysis methodology

Generalized Linear Models (GLM) with Poisson link were carried out for the 41 studied provinces, using rate of mortality due to COVID-19 (**CM**) per 1,000,000 inhabitants as the dependent variable and the air pollutants and meteorological variables as independent variables.

The GLM modeling process has two phases: on the one hand, the control of the trend, seasonalities and possible overdispersion of the series and, on the other hand, the possible effect that the atmospheric pollution and meteorological variables (with their corresponding lags) have on the dependent variable.

To control for the trend, a variable called n1 was used. This variable was defined as *n*1 = 1 for February 1, 2020; *n*2 = 2 for February 2, 2020 and so forth until the end of the period. To control for seasonalities of 4 months (120 days), 3 months (90 days), 2 months (60 days) and 1 month (30 days) the following variables were introduced:

sin120 = sin(2π*n1 (3/365.25); cosin120 = cosin(2π*n1 (3/365.25), sin90 = sin(2π*n1 (4/365.25), sin60 = sin(2π*n1 (6/365.25) and sin30 = sin(2π*n1 (12/365.25), in addition to the corresponding cosines of the same functions.

To control for possible overdispersion of the model, the autoregressive of order 1 of the dependent variable has been introduced.

GLM were carried out between the dependent variables and the average daily values of the independent variables, identifying lag days with significant differences. In the case of *T*max and HA, significant lags were considered, starting with the 5th day until the 28th day; one of the criteria considered was that temperature and humidity would be unable to aggravate the symptoms of the disease in an immediate way; it would be more likely that they could influence SARS-CoV-2 around the 5th day (incubation period). This criteria, coincides with a study that identified a greater correlation between temperature and COVID-19 after the 5th day of infection [6]. In contrast with the environmental variables, the air pollutants would be able to aggravate respiratory and circulatory system symptoms and cause a patent to seek health services to receive a diagnosis and be considered a case of COVID-19 on the same day [22]. For this reason, lags were not considered in the study of PM_10_ and NO_2_ between days 0 and 28.

A range of days was established for the analysis, lasting from 0 to 28 days, taking into account the beginning of symptoms through the time of death of the patient [36]. A model of weekly distribution of the lags was used [19, 38]. First, lags from 0 to 7 days were introduced, and later 8–14 days, 15–21 days and 22–28 days, maintaining significant lags until the completion of 28 days.

Using the estimators obtained from the Generalized Linear Models with Poisson Link (GLM), the relative risk (RR) were calculated, based on the formula RR = *e*^*β*^ where *β* is the absolute value of the estimator obtained. A negative value of the coefficient of the estimator indicates that an increase in the dependent variable is associated with a decrease in the independent variable. RR were calculated by an increase of 10 µg/m^3^ in PM_10_ and NO_2_, 1 ℃ in the case of *T*max and 1 g/m^3^ for HA. In order to better describe the results of RR associated with **CM**, maps were constructed that included the studied provinces; the information on risks is presented in terciles in natural breaks grouped in ascending order: RR terciles: tercile 1, tercile 2 and tercile 3. The attributable risk of some provinces was calculated based on the following formula: RR = (RR − 1)/RR.

In order to explain the heterogeneity in the geographical distribution of the results, a linear regression model was carried out. This model included the health determinants included in the Lalonde–Laframboise epidemiological model as independent variables (the Lalonde–Laframboise model) groups variables into lifestyle, environment, and health system response).

The equation is as follows: $\hat{Y} = \beta_{0} + \beta_{1} X_{1} + \beta_{2} X_{2} + \beta_{3} X_{3} + \ldots + \beta_{k} X_{k}$,where *Y* = risks associated with NO_2_, PM_10_, *T*max and HA by studied province, for each independent variable *X*_*i*_ the model considers a regression coefficient *βi*. This coefficient refers to the expected change in the dependent variable associated with a unit change in the corresponding independent variable (*Xi*: *X*1, *X*2, *X*3 o *Xk*), holding all of the other independent variables constant [20].

#### Software

SPSS 25.0 and Stata 16.0 software packages were used for the time series analysis. Maps were constructed using Qgis 3.16.3, and tables were produced using Excel.

### Results

Table 1 shows information on both the dependent variable (**CM**) and the lags where associations between the environmental variables considered and the dependent variable are established.

**Table 1:** Air pollutants and atmospheric variables associated with the COVID-19 mortality rate (**CM**) by lag days in provinces in Spain (^a^) from Feb. 1, to May 31, 2020

Spanish provinces	**CM**	COVID-19 mortality rate (**CM**) by associated lag days (Lag)			
		Air pollutants		Atmospheric variables	
		PM_10_	NO_2_	*T*max	HA
A Coruña	2.23	(–)	(–)	(5)	(–)
Alacant/Alicante	2.11	(–)	(–)	(–)	(5)
Albacete	10.77	(11)	(2, 5)	(18)	(6, 9, 23, 28)
Almería	0.64	(0,3,28)	(22,27)	(–)	(–)
Araba/Álava	9.02	(20)	(2, 11)	(8)	(6, 16, 19, 22)
Asturias	2.68	(2, 19, 23)	(–)	(–)	(–)
Ávila	10.96	(1, 4, 10, 19, 22, 25, 27)	(–)	(14, 20)	(–)
Barcelona	6.88	(–)	(–)	(–)	(21)
Bizkaia	6.10	(–)	(–)	(–)	(21)
Burgos	6.09	(21)	(–)	(14, 16, 24)	(20)
Cádiz	1.01	(21)	(–)	(–)	(–)
Cantabria	3.00	(2)	(20)	(–)	(–)
Castelló/Castellón	3.06	(–)	(16)	(20)	(14, 18, 28)
Ciudad Real	17.91	(26)	(6, 13, 18, 28)	(25)	(–)
Cuenca	12.56	(12, 19, 22, 27)	(28)	(7, 15, 24)	(5, 18, 25)
Gipuzkoa	3.29	(16)	(–)	(14)	(–)
Guadalajara	7.47	(2)	(8, 11, 14, 16)	(25)	(–)
Huelva	0.71	(18, 22)	(16)	(14)	(20, 24)
Illes Balears	2.31	(–)	(–)	(–)	(–)
La Rioja	8.79	(0, 10, 12, 21, 27)	(–)	(7, 14, 19)	(–)
León	7.17	(24)	(4, 7, 8, 12)	(–)	(19)
Lugo	0.79	(21, 24)	(8)	(20, 24)	(8, 14)
Madrid	10.30	(–)	(–)	(–)	(–)
Málaga	1.43	(23, 25)	(13, 28)	(–)	(–)
Murcia	0.79	(–)	(15, 25)	(–)	(–)
Navarra	6.59	(2, 3, 15)	(–)	(–)	(–)
Ourense	3.42	(–)	(2, 7, 10)	(18)	(14)
Palencia	5.67	(25, 27)	(9)	(19, 24, 27)	(23)
Pontevedra	1.32	(–)	(21)	(25, 28)	(–)
Salamanca	12.36	(4, 7)	(4, 16, 22, 26)	(–)	(8)
Segovia	21.42	(20)	(3, 25)	(8)	(23, 24)
Sevilla	1.18	(–)	(–)	(–)	(–)
Soria	20.18	(9, 18, 21, 24)	(12)	(9, 19)	(13, 22, 24)
Tarragona	3.43	(5, 27)	(–)	(9, 15, 28)	(–)
Toledo	8.62	(–)	(0, 13, 16, 24)	(–)	(–)
València/Valencia	2.28	(27)	(–)	(–)	(27)
Valladolid	8.12	(3, 21)	(8, 14, 18)	(24)	(–)
Zamora	6.20	(20, 22)	(4, 7, 9, 11, 21)	(3)	(21, 26)
Zaragoza	6.01	(24, 27)	(14, 20)	(–)	(–)
Santa Cruz de Tenerife	0.96	(–)	(0, 13, 16, 19, 23)	(–)	(–)
Las Palmas	0.32	(–)	(–)	(–)	(–)

^a^Studied provinces: 41 of 52; some provinces were not studied due to incomplete information

*(–)* no association

The **CM** values range from the highest values found for Segovia, Soria and Ciudad Real to the lowest in the provinces of Las Palmas, Almería, Huelva and Murcia. It was found that, as a general rule, the highest **CM** values are found in the interior provinces of the peninsula.

The associations of atmospheric pollutants with mortality shown in Table 1 are positive in the sense that the higher the concentration of pollutants, the higher the number of cases. As a general rule, in relation to delays, they show two types of effects: some in the short term (Lags 0–7) and others in the long term (lags 15–28). For temperature and humidity, the associations found are negative (the lower the humidity and temperature, the greater the effect on **CM**) and, in general, only the effect appears in the long term.

**Fig. 1:**
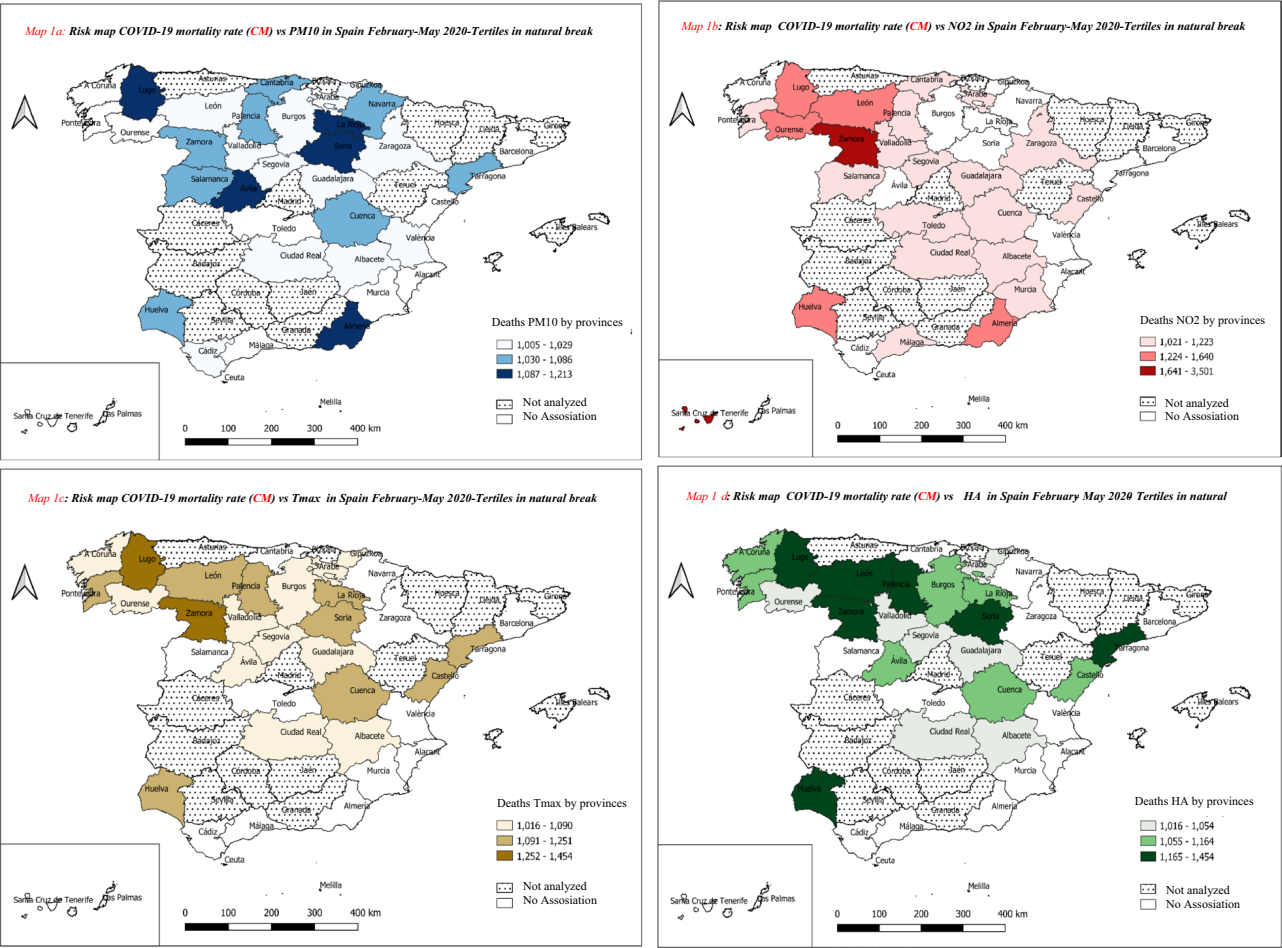
Risks of air pollutants and atmospheric variables associated with the COVID-19 mortality rate (**CM**) in provinces in Spain (*) from Feb. 1, to May 31, 2020. **a** Risk map COVID-19 mortality rate (**CM**) vs PM_10_ in Spain February–May 2020-tertiles in natural break; **b** risk map COVID-19 mortality rate (**CM**) vs NO_2_ in Spain February–May 2020-tertiles in natural break; **c** risk map COVID-19 mortality rate (**CM**) vs *T*max in Spain February–May 2020-tertiles in natural break; **d** risk map COVID-19 mortality rate (**CM**) vs HA in Spain February–May 2020-tertiles in natural break

Table 2 and Fig. 1a–d show the risks of the air pollutants and atmospheric variables associated with **CM**. Fig. 1a shows that the provinces that presented a greater number of RRs associated with PM_10_ are found in tercile 1 and tercile 3. The RRs of these terciles ranged between 1.030 and 1.213. Table 2 shows the AR by province; AR represents the contribution of air pollutants and atmospheric variables in the GLM. In the case of PM_10_ the province with the greatest RR associated with **CM** was Almeria, with an RR of 1.213 (1.026–1.400) and an AR of 18 percent. In other words, for each 10 g/m^3^ increase in PM_10_, the attributable risk (AR) of PM_10_ to **CM** was 18%.

**Table 2:** Relative risks (RR) associated with the COVID-19 mortality rate (**CM**) and air pollutants and atmospheric variables, by Spanish province (^a^) from Feb. 1, to May 31, 2020

Spanish provinces	**CM**	RRs Asociados a la tasa de mortalidad por COVID-19 (**CM**)							
		Air pollutants				Atmospheric variables			
		PM10	AR	NO_2_	AR	*T*max	AR	HA	AR
A Coruña	2.23	(–)	(–)	(–)	(–)	1.063 (1.010–1.115)	5.907	(–)	(–)
Alacant/Alicante	2.11	(–)	(–)	(–)	(–)	(–)	(–)	1.198 (1.044–1.352)	16.525
Albacete	10.77	1.020 (1.006–1.035)	2.003	1.118 (1.019–1.216)	10.532	1.037 (1.020–1.054)	3.609	1.630 (1.152–2.107)	38.634
Almería	0.64	1.213 (1.026–1.400)	17.552	1.352 (1.019–1.684)	26.013	(–)	(–)	(–)	(–)
Araba/Álava	9.02	1.019 (1.012–1.025)	1.835	1.047 (1.004–1.089)	4.455	1.036 (1.018–1.054)	3.489	1.725 (1.187–2.262)	42.014
Asturias	2.68	(–)	(–)	(–)	(–)	(–)	(–)	(–)	(–)
Ávila	10.96	1.135 (1.003–1.266)	11.877	(–)	(–)	1.081 (1.047–1.114)	7.454	(–)	(–)
Barcelona	6.88	(–)	(–)	(–)	(–)	(–)	(–)	1.082 (1.023–1.141)	7.557
Bizkaia	6.10	(–)	(–)	(–)	(–)	(–)	(–)	(–)	(–)
Burgos	6.09	1.013 (1.004–1.021)	1.240	(–)	(–)	1.090 (1.049–1.130)	8.232	1.181 (1.096–1.267)	15.358
Cádiz	1.01	1.016 (1.002–1.029)	1.530	(–)	(–)	(–)	(–)	(–)	(–)
Cantabria	3.00	1.042 (1.023–1.061)	4.032	1.046 (1.015–1.077)	4.400	(–)	(–)	(–)	(–)
Castelló/Castellón	3.06	(–)	(–)	1.047 (1.010–1.084)	4.500	1.123 (1.072–1.175)	10.989	1.707 (1.417–1.996)	41.402
Ciudad Real	17.91	1.006 (1.004–1.008)	0.594	1.132 (1.004–1.259)	11.625	1.028 (1.014–1.043)	2.769	(–)	(–)
Cuenca	12.56	1.044 (1.002–1.087)	4.257	1.021 (1.015–1.028)	2.089	1.124 (1.042–1.206)	11.015	1.331 (1.225–1.437)	24.864
Gipuzkoa	3.29	1.025 (1.008–1.041)	2.419	(–)	(–)	1.054 (1.022–1.086)	5.153	(–)	(–)
Guadalajara	7.47	1.016 (1.006–1.025)	1.535	1.151 (1.006–1.295)	13.095	1.034 (1.013–1.055)	3.308	(–)	(–)
Huelva	0.71	1.056 (1.001–1.111)	5.296	1.335 (1.076–1.593)	25.076	1.237 (1.057–1.417)	19.173	2.492 (2.109–2.875)	59.871
Illes Balears	2.31	(–)	(–)	(–)	(–)	(–)	(–)	(–)	(–)
La Rioja	8.79	1.144 (1.005–1.282)	12.555	(–)	(–)	1.131 (1.046–1.217)	11.612	(–)	(–)
León	7.17	1.007 (1.002–1.012)	0.697	1.395 (1.014–1.775)	28.307	1.183 (1.093–1.273)	15.492	(–)	(–)
Lugo	0.79	1.112 (1.003–1.221)	10.066	1.640 (1.129–2.151)	39.025	1.367 (1.278–1.457)	26.867	6.463 (2.928–9.999)	84.528
Madrid	10.30	(–)	(–)	(–)	(–)	(–)	(–)	(–)	(–)
Málaga	1.43	(–)	(–)	1.027 (1.006–1.048)	2.641	(–)	(–)	(–)	(–)
Murcia	0.79	(–)	(–)	1.114 (1.000–1.228)	10.239	(–)	(–)	(–)	(–)
Navarra	6.59	1.071 (1.000–1.142)	6.646	(–)	(–)	(–)	(–)	(–)	(–)
Ourense	3.42	(–)	(–)	1.530 (1.049–2.011)	34.641	1.040 (1.008–1.071)	3.824	1122 (1033–1212)	10.908
Palencia	5.67	1.047 (1.014–1.080)	4.496	1.120 (1.020–1.220)	10.722	1.220 (1.113–1.327)	18.026	1129 (1031–1228)	11.459
Pontevedra	1.32	(–)	(–)	1.051 (1.011–1.091)	4.838	1.164 (1.164–1.165)	14.103	(–)	(–)
Salamanca	12.36	1.045 (1.006–1.084)	4.295	1.223 (1.005–1.440)	18.211	(–)	(–)	1063 (0998–1128)	5.906
Segovia	21.42	1.005 (1.002–1.008)	0.496	1.082 (1.010–1.154)	7.574	1.016 (1.001–1.030)	1.547	1205 (1182–1228)	17.008
Sevilla	1.18	(–)	(–)	(–)	(–)	(–)	(–)	(–)	(–)
Soria	20.18	1.150 (1.007–1.293)	13.034	(–)	(–)	1.191 (1.115–1.266)	16.028	1736 (1326–2145)	42.380
Tarragona	3.43	1.086 (1.004–1.169)	7.953	(–)	(–)	1.251 (1.115–1.388)	20.082	(–)	(–)
Toledo	8.62	(–)	(–)	1.106 (1.010–1.202)	9.555	(–)	(–)	(–)	(–)
València/Valencia	2.28	1.006 (1.002–1.011)	0.625	(–)	(–)	(–)	(–)	1203 (1082–1324)	16.864
Valladolid	8.12	1.023 (1.003–1.043)	2.228	1.085 (1.006–1.165)	7.868	1.031 (1.011–1.051)	3.018	(–)	(–)
Zamora	6.20	1.064 (1.005–1.123)	6.002	3.501 (1.136–5.865)	71.435	1.454 (1.115–1.794)	31.238	1201 (1082–1319)	16.712
Zaragoza	6.01	1.029 (1.009–1.049)	2.818	(–)	(–)	(–)	(–)	(–)	(–)
Santa Cruz de Tenerife	0.96	(–)	(–)	1.927 (1.013–2.841)	48.097	(–)	(–)	(–)	(–)
Las Palmas de Gran Canarias	0.32	(–)	(–)	(–)	(–)	(–)	(–)	(–)	(–)

^a^Studied provinces: 41 of 52 Spanish provinces; some provinces were not studied due to incomplete information; RR is interpreted as an increase of 10 µg/m^3^ in PM_10_ and NO_2_, 1 ℃ in the case of Tmax and 1 g/m^3^ for HA

***CM*** COVID-19 mortality rate; *AR* attributable risk; *(–)* no association

Fig. 1b shows the map of risks of NO_2_ associated with **CM**. Four of seven provinces that belong to tercile 2 (RR:1.22–1.64) and tercile 3 (RR:1.64–3.50) are found in the northwest of Spain. The greatest effect is in the province of Lugo with an AR of 26.9%.

As can be seen both in Fig. 1a and b and in the results of the RRs shown in Tables 2 and 3, the effect of NO_2_ on **CM** occurs in a similar number of provinces (27 provinces with associations for PM_10_ compared to 24 provinces with associations for NO_2_), but, in general, the RRs found for PM_10_ are higher than those found for NO_2_.

**Table 3:** Percentage of air pollutants and atmospheric variables associated with the COVID-19 mortality rate (**CM**) in provinces in Spain (^a^) from Feb. 1, to May 31, 2020

	Association with air pollutants		Association with atmospheric variables	
	PM_10_	NO_2_	*T*max	HA
COVID-19 mortality rate				
Total Spanish provinces studied	41	41	41	41
Number of Spanish provinces with an association	27	24	21	18
% of Spanish provinces with an association	65.85	58.54	51.22	43.90

^a^Studied provinces: 41 of 52; some provinces were not studied due to incomplete information

***CM*** COVID-19 mortality rate

In our study, we identified a negative association between temperature and **CM** and HA and **CM**. In other words, with a lower temperature or lower HA, **CM** increases.

Fig. 1c shows the risks of temperature associated with **CM**. The provinces in tercile 3 (RRs of 1.25–1.45) that had a higher RR associated with temperature and **CM** were Lugo and Zamora, located in the northwest of Spain.

Fig. 1d shows the risks of HA associated with **CM**. Four of the seven provinces that belong to tercile 3 (RRs de 1.17–1.45) are found in the northwest of Spain.

The number of provinces where associations are found between MC and temperature (21) and with absolute humidity (18) is lower than those found for air pollutants.

The purpose of displaying the RR values found between MC and the different environmental variables on a map (Fig. 1) was to visualize the existence of differentiated geographical behavior in some regions compared to others.

The information from the risk maps is very heterogeneous, and does not contribute to delimiting areas with greater risk or geographical areas with common characteristics (hotspots) that would justify a greater or lesser distribution of the variables studied, which means that our analysis does not allow us to draw any important conclusions from a geographical point of view.

In order to explain the differences observed in the geographical distribution of the RRs at the country level (Fig. 1), other health determinants were analyzed (see Additional file 1: Table S2) using linear regression models. However, none of the explanatory variables considered was associated with **CM**.

## Discussion

Prior pandemics such as the 1918 flu (known as the Spanish Flu) and the pandemics of 1957 and 1968 resulted in millions of deaths worldwide [59]. These pandemics had an impact on mortality that was devastating for public health and for the world economy. The current COVID-19 pandemic is no exception. There have been a high number of deaths in Spain, thus it is important to determine which factors are related to mortality.

The results found in our study regarding higher **CM** values during the first wave in inland regions of the Peninsula are consistent with those found in other studies conducted in Spain and would indicate the important role that environmental variables played during the first wave, in the absence of pharmacological measures, in increasing temperature and absolute humidity [29]

In order to better understand the influence of the factors studied on **CM**, this discussion is divided into sections for each variable studied.

## PM_10_

This study showed a high percentage of provinces with an association between **CM** and PM_10_. It is known that in general, PM has a soluble fraction and an insoluble fraction that can act upon pulmonary cells and cause adverse effects [40]. The soluble fraction contains water, soluble ions and organic acids, while the insoluble fraction is primarily made up of kaolinite, calcium carbonate and organic carbon that is responsible for greater cellular mortality and severe cellular damage [69]. Furthermore, the exposure to PM may cause chronic inflammation and cellular damage through oxidative stress that causes alterations in the immune response, which makes a human being more susceptible to infections [61]. On the other hand, in analyzing the mechanism of action of PM on **CM**, some authors consider that pre-existing immune disorders brought about by exposure to high concentrations of PM_10_ and PM_2.5_ played an important role in the lethal nature of SAR-CoV-2 in Milán and Lombardy, Italy [23]. One study even found that PM_10_ presented a strong relationship with the number of deaths distributed in each Italian province affected by COVID-19 [16], and another study carried out in three French provinces suggested that an increase in PM_10_, generated an increase in **CM** [41]. It has also been observed that an increase in **CM** was associated with an increase in the concentration of PM_2.5_ in the United States [62]. Our results coincide with a study in China that found that increases in the concentrations of PM_10_ and PM_2.5_ were correlated with an increase in **CM** [67]. Although there are studies that mention a high correlation between PM_10_ and PM_2.5,_ PM_10_ has been independently associated with COVID-19 mortality specifically, such that it has come to be considered an independent predictor of mortality due to COVID-19 and is probably an early indicator of epidemic recurrence [55]. Along these lines, our study identified 66 percent of provinces associated with **CM** and PM_10_, which was the greatest percentage among the pollutants and atmospheric variables studied (see Table 3).

## NO_2_

The effect of NO_2_ on the aggravation of respiratory diseases has been shown above all in children [33]. There is scarce evidence among adults. On the other hand, studies of healthy volunteers have shown that environments with NO_2_ induce infiltration of inflammatory cells in airways [25]. Furthermore, it is believed that the exposure to pollutants such as NO_2_ could inhibit antimicrobial responses, reducing the elimination of the virus and promoting pulmonary infection [58]. In studies prior to the pandemic, it has been shown that acute exposure to NO_2_ causes oxidative stress and worsening of pulmonary function [26].

It has been suggested that patients with cardiovascular risks infected with SARS-CoV-2 could have higher levels of angiotensin 2 (ECA2), [34]. This could produce a disequilibrium in the anti-inflammatory response and contribute to a worsening of pulmonary function. Along these lines, one study established a possible role of NO_2_ in interfering with ECA2, due to the fact that a great quantity of ECA2 was identified in the epithelial cells of the lung after exposure to NO_2_ [4].

Studies related to COVID incidence have shown a greater association between COVID-19 incidence and NO_2_ in England ([58], and France [41]). In Spain, a study showed a short-term association between NO_2_ and the incidence and severity of COVID-19, but such an association was not found for **CM** [38]. Another study carried out by the European Space Agency (ESA) showed that 78% of deaths due to COVID-19 were concentrated in five areas in northern Italy and in central Spain, areas that also had high levels of NO_2_ [44]. A study in England found that long-term exposure to NO or NO_2_ was associated with an increase in **CM**, which confirms the findings at the regional and subregional level of associations between zones with greater concentrations of NO_2_ (above 100 µmol/m^2^) and zones with higher **CM** [58]. However, in our study, the analysis of long-term exposure to NO_2_ (see Additional file 1: Table S1) did not identify an association with **CM**.

The bibliography referenced in this discussion shows studies that link PMs with **CM**, and there are fewer studies that relate NO_2_ to **CM**. A study carried out in Queen County, New York (USA) identified a negative relationship between PM_2.5_ and **CM** [2]. When concentrations of PM_2.5_ were studied in the selected areas, the study confirmed that concentrations of PM_2.5_ were much lower than in the studies that found a greater association between **CM** and PM_2.5_ in Italy [24] and China [68]. The authors concluded that in these zones, other pollutants such as NO_2_ and SO_2_ could have had a much greater influence on the transmission and pathogenesis of COVID-19. This would suggest that in the places where PM concentrations are high, it is more likely that there is an association between **CM** and PM, and in places where PM concentrations are lower, other environmental pollutants could compete for an association with **CM**, which would explain some of the differences in the associations identified in some of the provinces studied in Spain. These studies agree with our findings of a greater number of provinces with an association between PM_10_ and **CM** (66%), followed by 58.54% of provinces with an association between **CM** and NO_2_.

## *T*max and HA

Although some authors link temperatures to greater virus transmission [37], a systematic review reveals that much of the evidence analyzed related to the association between temperature and the incidence of COVID-19 is considered low quality, due to defects in study design or missing information [42].

Prior to the pandemic, some authors considered that low levels of humidity could be an important risk factor for respiratory diseases, and that low humidity itself could cause a wide increase in the mortality rates due to respiratory diseases [5, 15] and could even increase mortality rates due to SARS [56]. At the beginning of the pandemic, some authors published articles stating that the transmission of SARS-CoV-2 was more efficient at lower temperatures and low humidity [51], based on laboratory studies in which SARS-CoV became inactive an higher temperatures and humidity [8]. However, the evidence around the relationship between temperature and **CM**, or the relationship between HA and **CM**, remains scarce. One study showed that an increase of one unit in humidity and in the range of daily daytime temperature was positively associated with a change of 0.28% and 2.22% in **CM**, respectively [47]. Another study in 166 countries identified a negative correlation between temperature and the number of deaths, and a negative relationship between relative humidity and the number of deaths [60–64]. In the present study we also identified a negative association, both for temperature and **CM** and for HA and **CM**. However, at the Spanish national level, both temperature and HA (atmospheric variables) presented a lower percentage of associated provinces, compared to the air pollutant variables (see Table 3). Therefore, the role played by atmospheric variables in **CM** is not yet clear.

## Geographical distribution and other health determinants

In order to explain the differences in the geographical distribution of the studied variables, we carried out an analysis of other health determinants based on the Lalonde–Laframboise model of health determinants (see Additional file 1: Table S1). However, we were not able to identify any health determinant associated with the GLM in the studied provinces. It could be that despite the fact that air pollutants and atmospheric variables are not sufficient alone to explain high **CM**, the determinants included in our model are not directly related to COVID-19 mortality. On the other hand, there are other variables that could be related to **CM**, for example sex, age, co-morbidities, and access to intensive care services, among others, which, due to the study design, were impossible to measure in sufficient detail.

### Conclusions

Our study identified atmospheric variables and air pollutants related to **CM**. Among the air pollutants, the role of PM_10_ is worth highlighting, as the chemical air pollutant for which there was a greater number of provinces in which it was associated with **CM**. The role of the meteorological variables—temperature and HA—was much less compared to that of the air pollutants.

None of the social determinants we proposed could explain the heterogeneous geographical distribution identified in this study. This is probably due to the fact that the variables to which we had access during our analysis are not the only variables that influence COVID-19 mortality. Mortality is also affected by factors such as access to mechanical fans, availability of emergency medical staff, pneumologists, and specialists in intensive care units, among other factors. Information on these factors was not available during the course of the analysis.

## Limitations

The methodology of the analysis of this is a descriptive observational study. Specifically, it is a population-based ecological study. Generally, in epidemiological studies it constitutes a basic level of evidence. This type of study does not allow for a causal relationship; but it constitutes a useful exploratory approach [49]. The study carried out by the authors corresponds to an ecological time series design, with all the epidemiological limitations inherent to this type of study [60], especially the ecological fallacy. The two previous points show the need for prudence when extrapolating the results to other temporal situations different from those corresponding to the moment of carrying out this study.

The period of confinement affected the exposure to pollutants [50, 52, 66] and to environmental variables in all of the provinces, above all in the provinces with a very low incidence. This decrease in air pollution levels as a consequence of confinement may affect the association that may exist between air pollution and COVID-19. However, it should be taken into account that the study design included the values of the atmospheric pollutants considered 28 days prior to the start of the confinement in Spain.

In addition, the lack of a polymerase chain reaction tests and its heterogeneous provincial distribution is an important bias that may condition the results of this study, especially in the incidence rate.

On the other hand, the environmental variables of exposure were not measured where the people who died were. The fact of using a single meteorological observatory or a few pollution measurement stations per province indicates that they cover very wide areas; which is associated with the Berkson type error [54].

## Supplementary Information

**Additional file 1: Table S1.** Independent variables that influence air pollutants and atmospheric variables included in the study, by the Lalonde Laframboise health determinants model, in Spanish provinces (*) from Feb. 1, to May 31, 2020.

## Data Availability

All data generated or analyzed during this study are included in this published article (“Methods” section) and publicly available dataset, the datasets used and/or analyzed during the current study are available from the corresponding author on reasonable request.

## Abbreviations

GLM: Generalized linear models
**CM**: Rate of mortality due to COVID-19
*T*max: Maximum daily temperature
HA: Average daily absolute humidity
NO_2_: Nitrogen dioxide
PM_10_: Particulate matter
HR: Average daily relative humidity
*T*: Average daily temperature
